# α‐Synuclein seed amplification assay detects Lewy body co‐pathology in autosomal dominant Alzheimer's disease late in the disease course and dependent on Lewy pathology burden

**DOI:** 10.1002/alz.13818

**Published:** 2024-04-26

**Authors:** Johannes Levin, Simone Baiardi, Corinne Quadalti, Marcello Rossi, Angela Mammana, Jonathan Vöglein, Alexander Bernhardt, Richard J. Perrin, Mathias Jucker, Oliver Preische, Anna Hofmann, Günter U. Höglinger, Nigel J. Cairns, Erin E. Franklin, Patricio Chrem, Carlos Cruchaga, Sarah B. Berman, Jasmeer P. Chhatwal, Alisha Daniels, Gregory S. Day, Natalie S. Ryan, Alison M. Goate, Brian A. Gordon, Edward D. Huey, Laura Ibanez, Celeste M. Karch, Jae‐Hong Lee, Jorge Llibre‐Guerra, Francisco Lopera, Colin L. Masters, John C. Morris, James M. Noble, Alan E. Renton, Jee Hoon Roh, Matthew P. Frosch, C. Dirk Keene, Catriona McLean, Raquel Sanchez‐Valle, Peter R. Schofield, Charlene Supnet‐Bell, Chengjie Xiong, Armin Giese, Oskar Hansson, Randall J. Bateman, Eric McDade, Dominantly Inherited Alzheimer Network, Piero Parchi

**Affiliations:** ^1^ Department of Neurology LMU University Hospital, LMU Munich Munich Germany; ^2^ German Center for Neurodegenerative Diseases Munich Germany; ^3^ Munich Cluster for Systems Neurology (SyNergy) Munich Germany; ^4^ Department of Biomedical and Neuromotor Sciences University of Bologna Bologna Italy; ^5^ IRCCS Istituto delle Scienze Neurologiche di Bologna Bologna Italy; ^6^ Department of Pathology and Immunology Washington University School of Medicine Saint Louis Missouri USA; ^7^ Department of Neurology Washington University School of Medicine Saint Louis Missouri USA; ^8^ German Center for Neurodegenerative Diseases (DZNE) Tübingen Germany; ^9^ Hertie Institute for Clinical Brain Research University of Tübingen Tübingen Germany; ^10^ Living Systems Institute Faculty of Health and Life Sciences University of Exeter Exeter UK; ^11^ FLENI, Montañeses 2325 (C1428AQK) Buenos Aires Argentina; ^12^ Department of Psychiatry Washington University School of Medicine Saint Louis Missouri USA; ^13^ University of Pittsburgh Neurology Pittsburgh Pennsylvania USA; ^14^ Department of Neurology Massachusetts General Hospital, Harvard Medical School Boston Massachusetts USA; ^15^ Department of Neurology Mayo Clinic in Florida Jacksonville Florida USA; ^16^ Dementia Research Centre Department of Neurodegenerative Disease UCL Queen Square Institute of Neurology London UK; ^17^ UK Dementia Research Institute at UCL London UK; ^18^ Department of Genetics & Genomic Sciences Icahn School of Medicine at Mount Sinai New York New York USA; ^19^ Butler Hospital Brown Center for Alzheimer's Disease Research Alpert Medical School of Brown University Providence Rhode Island USA; ^20^ Department of Neurology Asan Medical Center Seoul South Korea; ^21^ Grupo de Neurosciencias de Antioquia, Sede de Investigación Universitaria SIU Medellín Colombia; ^22^ Florey Institute and The University of Melbourne Melbourne Victoria Australia; ^23^ Department of Neurology Taub Institute for Research on Alzheimer's Disease and the Aging Brain, and GH Sergievsky Center, Columbia University New York New York USA; ^24^ Department of Genetics and Genomic Sciences and Nash Family Dept of Neuroscience Icahn School of Medicine at Mount Sinai New York New York USA; ^25^ Departments of Neurology and Physiology Korea University College of Medicine Seoul South Korea; ^26^ MassGeneral Institute for Neurodegenerative Diseases, Neuropathology Service, Massachusetts General Hospital Boston Massachusetts USA; ^27^ Department of Laboratory Medicine and Pathology University of Washington Seattle Washington USA; ^28^ Department of Anatomical Pathology AlfredHealth Melbourne Victoria Australia; ^29^ Alzheimer's Disease and Other Cognitive Disorders Unit, Service of Neurology, Hospital Clinic de Barcelona, FRCB‐IDIBAPS Barcelona Spain; ^30^ Neuroscience Research Australia Sydney New South Wales Australia; ^31^ School of Medical Sciences University of New South Wales Sydney New South Wales Australia; ^32^ Division of Biostatistics Washington University School of Medicine Saint Louis Missouri USA; ^33^ Modag GmbH Wendelsheim Germany; ^34^ Clinical Memory Research Unit Department of Clinical Sciences Malmö Faculty of Medicine, Lund University Lund Sweden; ^35^ Memory Clinic Skåne University Hospital Lund Sweden

**Keywords:** alpha‐synuclein seed amplification assay, Dominantly Inherited Alzheimer Network, Lewy body pathology, real‐time quaking‐induced conversion

## Abstract

**INTRODUCTION:**

Amyloid beta and tau pathology are the hallmarks of sporadic Alzheimer's disease (AD) and autosomal dominant AD (ADAD). However, Lewy body pathology (LBP) is found in ≈ 50% of AD and ADAD brains.

**METHODS:**

Using an α‐synuclein seed amplification assay (SAA) in cerebrospinal fluid (CSF) from asymptomatic (*n* = 26) and symptomatic (*n* = 27) ADAD mutation carriers, including 12 with known neuropathology, we investigated the timing of occurrence and prevalence of SAA positive reactivity in ADAD in vivo.

**RESULTS:**

No asymptomatic participant and only 11% (3/27) of the symptomatic patients tested SAA positive. Neuropathology revealed LBP in 10/12 cases, primarily affecting the amygdala or the olfactory areas. In the latter group, only the individual with diffuse LBP reaching the neocortex showed α‐synuclein seeding activity in CSF in vivo.

**DISCUSSION:**

Results suggest that in ADAD LBP occurs later than AD pathology and often as amygdala‐ or olfactory‐predominant LBP, for which CSF α‐synuclein SAA has low sensitivity.

**Highlights:**

- Cerebrospinal fluid (CSF) real‐time quaking‐induced conversion (RT‐QuIC) detects misfolded α‐synuclein in ≈ 10% of symptomatic autosomal dominant Alzheimer's disease (ADAD) patients.
- CSF RT‐QuIC does not detect α‐synuclein seeding activity in asymptomatic mutation carriers.
- Lewy body pathology (LBP) in ADAD mainly occurs as olfactory only or amygdala‐predominant variants.
- LBP develops late in the disease course in ADAD.
- CSF α‐synuclein RT‐QuIC has low sensitivity for focal, low‐burden LBP.

## 1 INTRODUCTION

Autosomal dominant Alzheimer's disease (ADAD) is a rare form of Alzheimer's disease (AD) that arises from mutations in the genes encoding presenilin 1 (*PSEN1*), presenilin 2 (*PSEN2*), or amyloid precursor protein (*APP*), all affecting APP processing.^1^ The hallmarks of sporadic AD (sAD) and ADAD are the accumulation of extracellular amyloid beta plaques and the aggregation of hyperphosphorylated tau proteins inside neurons, leading to the progressive loss of synapses and neurons.^2^ However, AD brains often exhibit co‐pathologies, including other abnormal protein aggregates, especially misfolded α‐synuclein (α‐syn), forming intraneuronal Lewy bodies (LBs) and Lewy neurites.^3^ With increased frequency compared to age‐matched non‐AD individuals, LB pathology (LBP) has been documented *post mortem* in 31% to 54% of sAD and 27% to 85% of ADAD patients’ brains, with significant variability among studies.^4^, ^5^, ^6^, ^7^, ^8^, ^9^, ^10^, ^11^, ^12^

In AD brains, especially those with ADAD^8^ and early onset sAD, α‐syn immunoreactivity is often predominantly or exclusively detected in the amygdala with limited associated accumulation in other limbic areas and in the brainstem.^4^, ^6^, ^7^, ^11^ These findings have been referred to as the amygdala‐predominant variant of LBP (Amg‐LBP) in which α‐syn accumulation does not follow the typical pattern of topographic distribution associated with Parkinson's disease (PD) and dementia with Lewy bodies (DLB) described by the Braak staging.^13^ The strong link with AD and the particular anatomical distribution suggest that Amg‐LBP might occur secondarily to AD pathology, the latter modulating the susceptibility of different brain regions to LBP.

Until recently, our knowledge of LBP in AD came from *post mortem* studies. Consequently, the mechanisms underlying the connection between AD and LB pathologies, the timing of α‐syn accumulation, and the specificities of α‐syn–related pathology in ADAD compared to sAD are poorly understood.

The development of seed amplification assays (SAAs) of misfolded α‐syn has recently provided a robust in vivo biomarker for LBP.^14^, ^15^ SAAs have demonstrated high specificity and sensitivity in detecting pathological α‐syn seeds in the cerebrospinal fluid (CSF) of patients with PD and DLB, even during the prodromal (i.e., isolated rapid eye movement sleep behavior disorder or mild cognitive impairment) or preclinical stage.^16^, ^17^, ^18^, ^19^, ^20^

This study aimed to investigate the presence and timing of SAA reactivity to detect LBP in ADAD in vivo. Initially, we investigated the presence of LBP via SAA examinations of CSF in local cohorts of the Dominantly Inherited Alzheimer Network (DIAN) in Germany, including living asymptomatic and symptomatic ADAD patients as well as healthy controls. Next, we tested the CSF of ADAD cases in a cohort with *post mortem* semiquantitative assessment of LBP.

## 2 METHODS

### 2.1 Study participants

The first part of this study involved participants from Munich and Tübingen DIAN study sites, including mutation non‐carriers (*n *= 29), asymptomatic (*n* = 26), and symptomatic (*n* = 15) mutation carriers. Asymptomatic individuals were defined by having a global Clinical Dementia Rating (CDR) score = 0, whereas symptomatic by a CDR score > 0. The second part of the study included 12 symptomatic individuals with neuropathological information available in addition to the core DIAN dataset. All these cases were evaluated by the DIAN observational study (DIAN‐Obs, data freeze number 16), and their CSF was provided by the DIAN‐Obs biorepository (Washington University, St. Louis, Missouri, USA). All cases with neuropathology were evaluated by the DIAN‐Obs Neuropathology Core (Washington University, St. Louis, Missouri, USA). Among the symptomatic individuals, two patients, labelled “converters,” were asymptomatic at baseline DIAN assessment and became symptomatic (CDR > 0) during follow‐up. In this context, baseline is defined as the first clinical assessment with CSF sampling.

### 2.2 Ethics approval

DIAN received approval from the ethics committees of Ludwig–Maximilians–University Munich and Eberhardt Karls University Tübingen (371‐13; 535/2011BO1, respectively), as well as the institutional review board committee of Washington University in St. Louis, Missouri, USA (201106339). In addition, permission to perform these measurements was obtained from the ethics committee of Ludwig–Maximilians‐University (371‐13). All participants provided written informed consent for CSF donation for research purposes; similarly, brain donations were obtained after acquiring written informed consent from participants and/or their legal representatives in accordance with applicable local laws and practices.

**RESEARCH IN CONTEXT**

1. **Systematic review**: The authors reviewed the literature using online resources such as PubMed, Web of Science, and Scopus. We found several studies applying the α‐synuclein (α‐syn) seed amplification assay (SAA) to detect Lewy body pathology (LBP) in vivo in patients with Parkinson's disease, dementia with Lewy bodies and sporadic Alzheimer's disease (sAD), including prodromal syndromes. However, we did not find publications on autosomal dominant Alzheimer's disease (ADAD).
2. **Interpretation**: In this study, we investigated the timing of occurrence and prevalence of misfolded α‐syn positivity as evidence of LBP, which is a frequent *post mortem* finding in AD. Our findings suggest that in ADAD, LBP occurs later than AD pathology and often as amygdala‐ or olfactory‐predominant LBP, for which cerebrospinal fluid α‐syn SAA has low sensitivity.
3. **Future directions**: Future studies should expand the in vivo analysis of LBP by α‐syn SAA to larger cohorts of ADAD patients, stratified for the type of mutation. Moreover, the prevalence of LBP detected by α‐syn SAA should be determined in sporadic early‐onset AD. Finally, the kinetic properties of brain misfolded α‐syn should be compared between individuals with typical LBD and those with the amygdala‐predominant variant.

### 2.3 CSF collection and analysis

CSF samples were collected by lumbar puncture (LP) from living individuals and processed as previously described.^1^ CSF α‐syn SAA (i.e., real‐time quaking‐induced conversion assay [RT‐QuIC]), including the recombinant wild‐type human α‐syn purification, was performed as previously described,^16^ with minor[Table alz13818-tbl-0001], [Table alz13818-tbl-0002] modifications. We ran the same positive and negative control samples throughout all experiments to optimize the comparison among fluorescent signal responses in different plates. As positive controls, we used samples from two patients with normal pressure hydrocephalus for whom a large CSF volume was available, who consistently showed a 4 of 4 positive wells response over at least 10 consecutive runs, indicating a high α‐syn seeding activity. Conversely, the negative controls were CSF from individuals with no clinical evidence of neurodegenerative disease consistently displaying a negative RT‐QuIC response (0 of 4). To overcome batch‐to‐batch variations and intrinsic plate‐to‐plate variability, we normalized the relative fluorescent units for every time point to the median of the maximum intensity (Imax) reached by four positive control replicates within each plate and expressed it as a percentage. We then set the threshold at 20% of the above parameter and the cut‐off at 30 hours. The rationale of the 20% choice stems from the observation that the samples expected to be negative (e.g., CSF from patients lacking LB pathology at neuropathologic examination) sometimes show a slight increase in the fluorescence signal toward the end of the run (between 20 and 30 hours). Therefore, reducing this threshold below the value of 20% would have increased the risk of having false positive readings. CSF samples were deemed positive when at least two of four replicates crossed the threshold as described.^16^

All CSF α‐syn SAA analyses were carried out at the Neuropathology Laboratory of the IRCCS Institute of Neurological Science of Bologna, Italy, by personnel blinded to the participants’ clinical or genetic status.

### 2.4 *Post mortem* neuropathology

Neuropathologic assessment[Table alz13818-tbl-0003] included a systematic evaluation of the left hemibrain by experienced neuropathologists (RJP and NJC) according to an established protocol^8^ and National Institute on Aging–Alzheimer's Association consensus criteria for AD neuropathological changes.^21^ LBP was assessed by immunohistochemistry using an antibody against phosphorylated α‐syn (Phospho‐α‐synuclein[Ser129], Cell Applications).

LBs were scored semi‐quantitatively according to McKeith et al.’s^22^ criteria (—= none; + = < 1 LB inclusion per 10x objective field; ++ = 1–3 LBs; +++ = 4–10 LBs; ++++ = > 10 or numerous LBs) in the following regions: medulla oblongata, locus coeruleus, pontine tegmentum, substantia nigra, basal forebrain including nucleus basalis, olfactory cortex (with olfactory tract and peduncle when available), striatum, thalamus, pallidum, dentate gyrus, hippocampal areas CA4‐CA2, CA1, subiculum, parahippocampal gyrus, fusiform gyrus, amygdala, anterior entorhinal cortex, anterior cingulate gyrus, superior and middle temporal gyri, middle frontal gyrus, precentral gyrus (when available), inferior parietal lobule, and occipital cortex. Where LBs were rare or absent, Lewy neurites/grains were scored separately from LBs.

For each case, the stage of LBP was classified according to Braak criteria for PD^13^ and McKeith criteria for DLB only in cases with brainstem involvement.^23^ Other cases, as appropriate, were classified as Amg‐LBP^6^ or simply described by area(s) of involvement.

### 2.5 Statistical analyses

Values for continuous parameters were expressed as means ± standard deviations; categorical parameters, as absolute values (%) or as median and interquartile range. The Student *t* test or Mann–Whitney *U* test were used to compare continuous parameters. The chi‐squared test was used for the number of consecutive SAA assessments per participant.

## 3 RESULTS

There were no significant differences in demographic features between asymptomatic mutation carriers and non‐carriers (Table 1). All CSF α‐syn SAA assessments in the asymptomatic cohort were negative.

**TABLE 1 alz13818-tbl-0001:** Characteristics of the asymptomatic study participants from the Munich & Tübingen DIAN cohorts at baseline α‐syn SAA assessments and numbers of consecutive α‐syn SAA assessments per participant.

	Asymptomatic mutation carriers (*n* = 26)	Mutation non‐carriers (*n* = 29)	*P* value
Age, years (SD)	35.2 (10.1)	34.6 (9.3)	0.82
Parental AAO, median years (interquartile range)	53 (47.0–56.3)	48 (40.5–52.5)	0.14
EYO (SD)	−16.6 (9.0)	N/A	N/A
Participants with 1/2/3/4 consecutive α‐syn SAA assessments, n	8/9/4/5	11/13/4/1	0.29

Abbreviations: AAO, age at onset; DIAN, Dominantly Inherited Alzheimer Network; EYO, estimated years from symptom onset; N/A, not applicable; SD, standard deviation; α‐syn SAA, α‐synuclein seed amplification assay.

Details on the symptomatic ADAD participants, including the mutated genes, are reported in Table 2. The results obtained in the symptomatic/converter cohorts are shown together (*n* = 27, of which *n* = 15 were DIAN participants from Munich and Tübingen, and *n* = 12 were ADAD individuals who underwent neuropathological assessment). The mean age of symptom onset was 43 ± 7.8 years. The range between age at onset and age at first LP was 1 to 8 years (Table 2), and the interval from the last LP to death in the autopsy‐confirmed cases ranged from 0.7 to 5.7 years (Table 3, Figure S1 in supporting information).

**TABLE 2 alz13818-tbl-0002:** Characteristics of the symptomatic study participants and converters at baseline α‐syn SAA assessment, numbers of consecutive α‐syn SAA assessments per participant, and information if an autopsy was performed.

Participant #	α‐syn SAA result	Consecutive α‐syn SAA assessments, [n]	Sex	Time point of first and last LP relative to symptom onset, [years]	Affected gene	Autopsy
	Symptomatic participants					
1	Positive	1	m	7	*PSEN1*	no
2	Positive	4	m	5, 7	*APP*	no
3	Positive	1	m	7	*PSEN1*	yes
4	Negative	1	m	2	*PSEN1*	no
5	Negative	1	m	7	*PSEN1*	no
6	Negative	1	m	4	*PSEN1*	no
7	Negative	1	f	3	*PSEN1*	no
8	Negative	3	m	3, 4	*APP*	no
9	Negative	4	f	3, 8	*PSEN1*	no
10	Negative	4	m	3, 8	*PSEN1*	no
11	Negative	3	m	0, 3	*APP*	no
12	Negative	2	f	2, 3	*PSEN1*	no
13	Negative	2	f	5, 6	*APP*	no
14	Negative	2	f	5, 6	*PSEN1*	no
15	Negative	2	m	4, 6	*APP*	no
16	Negative	1	m	6	*PSEN1*	yes
17	Negative	4	m	1, 4	*PSEN1*	yes
18	Negative	2	f	2, 3	*PSEN1*	yes
19	Negative	4	f	2, 7	*PSEN1*	yes
20	Negative	2	f	1, 2	*PSEN1*	yes
21	Negative	1	m	3	*PSEN1*	yes
22	Negative	2	m	7, 8	*APP*	yes
23	Negative	2	f	2,3	*PSEN1*	yes
24	Negative	1	f	8	*PSEN1*	yes
25	Negative	1	m	5	*PSEN1*	yes
	Converters					
26	Negative	2	f	–1, 1	*APP*	no
27	Negative	2	f	–1, 2	*PSEN1*	yes

Abbreviations: *APP*, gene encoding the amyloid precursor protein; f, female; LP, lumbar puncture; m, male; *PSEN1*, gene encoding presenilin 1; α‐syn SAA, α‐synuclein seed amplification assay.

**TABLE 3 alz13818-tbl-0003:** Characteristics of Lewy body pathology in the most representative areas of the autopsied study participants.

Participant, #	Time CSF collection—autopsy (years)^a^	α‐syn immuno‐reactivity	Distribution of Lewy body pathology	Burden of Lewy body pathology by area							
				OLF ctx	ME/LC	SN	CA1/subic	ENT ctx	Amg	aC	NEO ctx
3	0.7	Yes	Diffuse, mild in brainstem	++++	+	+	+++	+++	++++	+	+^˟^
16	2.4	Yes	Olfactory only	+++	–	–	–	–	–	–	–
17	3.1	No	–	–	–	–	–	–	–	–	–
18	0.6	Yes	Brainstem/limbic	+/‐	–	+++	–	+/‐	++	+/‐	–
19	1.1	Yes	Amygdala predominant	++	–	–	++	–	+++	–	–
20	5.7	No	–	n.a.	–	–	–	–	–	–	–
21	2.4	Yes	Amygdala/limbic predominant	++	–	+	+	+++	+++	+	–
22	2.2	Yes	Amygdala predominant	+++	–	–	–	++	+++	–	–
23	2.9	Yes	Olfactory only	[+]	–	–	–	–	–	–	–
24	3.1	Yes	Olfactory only	++	–	–	–	–	–	–	–
25	4.9	Yes	Amygdala only	–	–	–	–	–	+/++	–	–
27	1.4	Yes	Rare sparse Lewy pathology	n.a.	–	[+]	–	–	–	+	–

*Note*: α‐syn immunoreactivity burden was scored as follows :— = none; +/– = very rare (< 3 LB in the area); + = rare/sparse (< 1 LB inclusion per x10 field); ++ = moderate (1–3 LBs); +++ = frequent (4–10 LBs); ++++ = numerous (> 10 LBs).

Abbreviations: aC, anterior cingulate cortex; Amg, amygdala; CA1, cornus ammonis sector 1 (hippocampus); CSF, cerebrospinal fluid; EC, entorhinal cortex; LBs, Lewy bodies; LC, locus coeruleus; ME, medulla oblongata; n.a., not available; NEO ctx, neocortex; OLF ctx, olfactory cortex; SN, substantia nigra; subic, subiculum.

^a^ We indicated the last lumbar puncture in cases undergoing multiple assessments.

^˟^ Represents an average score (from—or + in most cortical areas to +++ in superior temporal gyrus). Score in square brackets indicates that only Lewy neurites were detected.

The CSF α‐syn SAA was positive in 3/27 (11.1%) symptomatic participants. In one of them (participant #2), each of four consecutive CSF samples taken longitudinally during 4 years of follow‐up starting from 4 years after symptom onset gave a positive result. All six SAA‐positive CSF samples (i.e., three patients at baseline plus three longitudinal analyses in one individual) showed α‐syn seeding activity in all tested replicates (i.e., 4 of 4). Compared to the positive controls, the positive ADAD patients showed a longer time to the threshold (Lag phase) and a tendency toward a lower Imax, suggesting a lower seeding activity (Figure S2 in supporting information). Both clinical converters to mild cognitive impairment tested negative by CSF α‐syn SAA at baseline (asymptomatic stage) and at 2 and 1 years after the development of cognitive decline.

**FIGURE 1:**
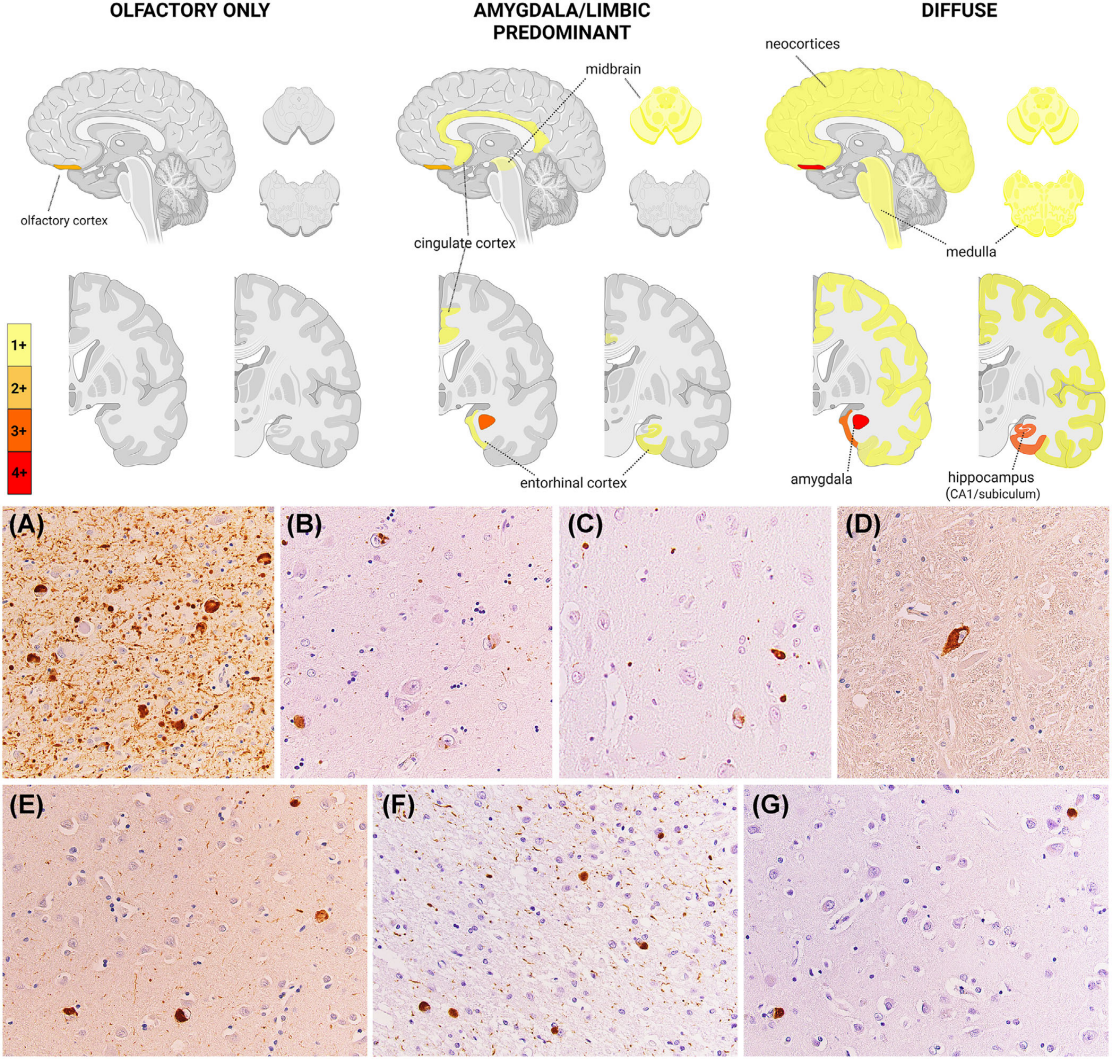
Representative LB pathology features in ADAD. The upper panel shows brain maps of LB pathology distribution in the three representative profiles (i.e., olfactory only, amygdala/limbic predominant, and diffuse) observed in the ADAD individuals at neuropathologic examination. Color scale of the brain maps represents the mean semiquantitative scores in each profile. The lower panel shows LBP features in the ADAD brains. A–C, Severe to moderate/mild LB and neuritic pathology in the amygdala (A, LBP ++++, case #3; B, LBP +++, case #21; C, LBP ++, case #18). D, Isolated syn immunoreactivity in a neuron of the medullary raphe, and (E) moderate LBP in the neocortex of the superior temporal gyrus in the only brain with diffuse LBP (case #3). F, Abundant Lewy neurites and LB in the olfactory tract, and (G) scattered LBs in the olfactory cortex of case #18. Magnification is x40 for all micrographs. ADAD, autosomal dominant Alzheimer's disease; LB, Lewy bodies; LBP, Lewy body pathology.

Of the 12 brain donors, all of whom had developed high levels of AD neuropathologic changes, 10 (83%) also displayed LBP (Table 3, Figure 1). However, only one of them (10%) showed positive seeding activity by CSF α‐syn SAA (Table 3). The brain of the α‐syn SAA positive donor (participant #3) showed the highest burden of LBP, and was the only one with neocortical LBP (i.e., Braak stage 6; diffuse neocortical stage according to McKeith et al.,^23^ although with an atypical distribution due to the mild involvement of the brainstem). Time between LP and death in this individual was relatively short, that is, 0.7 years. Among the other 11 participants with neuropathologic data, all with negative α‐syn SAA results, one had substantial LBP within the amygdala and substantia nigra (participant #18), four (33.3%) showed the Amg‐LBP variant pattern (#19, 21, 22, 25), three (25%) had immunoreactivity restricted to the olfactory area (piriform cortex, olfactory tract, and peduncle; participants #16, 23, 24), two were negative (#17 and 20) and one virtually negative (#27).

The four individuals with Amg‐LBP showed mild to moderate LBP involving only the amygdala (*n* = 1) or the amygdala together with other limbic structures, such as the entorhinal cortex and the hippocampus, with sparse or no brainstem involvement (Table 3). The time between LP and death in these four participants was 1.4, 2.4, 3.1, and 5.7 years, respectively.

Notably, 53 out of 82 participants (64.6%) underwent at least two consecutive α‐syn SAA assessments using material of independent LPs. All intraindividual consecutive α‐syn SAA assessments showed the same result as at baseline, highlighting the complete consistency of results within each participant.

## 4 DISCUSSION

The results of the present study, combined with those recently obtained in sAD,^20^, ^24^ shed new light on the association and pathogenetic interaction between LBP and AD pathology in the symptomatic phase of AD. The significantly lower percentage of LBP we detected via SAA in CSF of symptomatic patients with ADAD compared to sAD patients (11% vs. 21%) suggests a lower prevalence of “typical” LBP (i.e., following Braak staging) in ADAD compared to sAD most likely due to the significantly lower mean age of patients with symptomatic ADAD compared to those with sAD. Indeed, a positive association between age and “typical” LBP has been documented in both non‐AD and AD‐affected brains, especially between the presenile and senile age.^11^, ^25^

In contrast to “typical” LBP, the Amg‐LBP variant, which almost only affects patients with AD pathology, suggesting a secondary synucleinopathy, is more common in patients with early‐onset AD than in patients with late‐onset AD.^11^ Consistent with these previously published data, 4 of the 12 ADAD participants in our neuropathologically verified cohort showed LBP compatible with Amg‐LBP, whereas only one had diffuse LBP reaching the neocortical areas. The low overall detection sensitivity of the α‐syn SAA for LBP in this ADAD cohort is in line with the previously reported reduced sensitivity of these assays for the Amg‐LBP variants and other conditions in which the LBP is only focally present (e.g., limited to the lower brainstem or the olfactory areas) compared to the virtually full sensitivity in cases with a neocortical or limbic stage of LB disease pathology.^26^, ^27^, ^28^ In line with these findings, by applying the same SAA protocol to a cohort of 59 CSF and brain pairs, we have recently confirmed the 100% sensitivity of our assay in detecting the neocortical and limbic LBP stages.^28^ One might argue that the 100% sensitivity in “typical” LBD at the limbic stage compared to the negative finding we obtained in this study in the two participants with involvement of the limbic areas might represent a discrepancy. However, there is a profound difference in the LBP load/burden between the limbic stage of typical LBD, characterized by prominent involvement (corresponding to a score +++ / ++++ of the present study) of the brainstem (and hypothalamus/basal forebrain as well) and the ADAD participant with limbic predominant pathology showing minimal or only focal pathology in those areas. Consequently, the different overall burden of LB pathology might be sufficient to explain the different sensitivity of our α‐syn SAA between the two patient groups. However, we cannot entirely dismiss the hypothesis that Amg‐LBP in ADAD could be due to a different strain of α‐syn showing a lower SAA reactivity as it was demonstrated for multiple system atrophy.^16^, ^28^, ^29^

The range of intervals between CSF assessment and death in our cohort may also have contributed to the low positivity rate of α‐syn SAA in this study. Indeed, given the focal and sparse nature of the LBP in many of our patients with *post mortem* evaluation, it is plausible to believe that some patients were free of misfolded α‐syn deposition at the time of LP. The secondary nature of the Amg‐LBP variant, likely triggered or strongly modulated by AD pathology, also fits the idea of LBP being a relatively late event in most ADAD brains.

A limitation of the present study concerns the low number of individuals with positive α‐syn SAA not allowing a stratification according to mutated gene (e.g., *PSEN1* vs. *APP*). Moreover, the mean estimated years to symptom onset (EYO) of −16.6 years in the cohort of asymptomatic ADAD individuals suggests that in many cases, the amyloid deposition was in very early stages as the amyloid deposition typically starts ≈ −20 EYO.^30^ Finally, the timing between LP and death was heterogeneous in the cohort with *post mortem* neuropathology.

In summary, in this relatively small but comprehensive cohort of patients with ADAD, including asymptomatic carriers and symptomatic patients with or without *post mortem* neuropathology, we show that LBP can be detected as a CSF biomarker in symptomatic mutation carriers but at a lower rate than in sAD. This is likely due to the lower incidence of “typical” transitional/limbic and neocortical/diffuse LBP stages in presenile patients and the focal and late occurring nature of Amg‐LBP in ADAD.

While the clinical consequences of LBP in ADAD remain to be fully understood, analyzing CSF biomarkers of LBP in vivo could provide a better precision‐medicine approach for the clinical management of ADAD patients and for designing and interpreting data from disease‐modifying drug trials.

## CONFLICT OF INTEREST STATEMENT

JL reports speaker fees from Bayer Vital, Biogen, EISAI, Merck, Roche, TEVA, and Zambon; consulting fees from Axon Neuroscience, EISAI, and Biogen; author fees from Thieme medical publishers and W. Kohlhammer GmbH medical publishers; and is inventor in a patent “Oral Phenylbutyrate for Treatment of Human 4‐Repeat Tauopathies” (EP 23 156 122.6) filed by LMU Munich. In addition, he reports compensation for serving as chief medical officer for MODAG GmbH; is beneficiary of the phantom share program of MODAG GmbH; and is inventor in a patent “Pharmaceutical Composition and Methods of Use” (EP 22 159 408.8) filed by MODAG GmbH, all activities outside the submitted work. NCF reports consulting fees from Biogen, Eisai, Ionis, Lilly, Roche/Genentech, and Siemens all paid to UCL; he has also served on a Data Safety Monitoring Board for Biogen. OH has acquired research support (for the institution) from ADx, AVID Radiopharmaceuticals, Biogen, Eli Lilly, Eisai, Fujirebio, GE Healthcare, Pfizer, and Roche. In the past 2 years, he has received consultancy/speaker fees from AC Immune, Amylyx, Alzpath, BioArctic, Biogen, Cerveau, Eisai, Eli Lilly, Fujirebio, Merck, Novartis, Novo Nordisk, Roche, Sanofi, and Siemens. FL has research support from NIA, NIH, Biogen, Tau‐Consortium, Roche and is a consultant of Viewmind and Biogen. JCM is funded by NIH grants # P30 AG066444; P01AG003991; P01AG026276. Neither Dr. Morris nor his family owns stock or has equity interest (outside of mutual funds or other externally directed accounts) in any pharmaceutical or biotechnology company. AMG serves on SABs for Genentech and Muna Therapeutics and has received consultancy/speaker fees from Biogen. RSV reports consultancy or speaker fees from Ionis, AviadoBio, NovoNordisk, Pfizer, Neuraxpharm, and Roche diagnosis. GH has ongoing research collaborations with Roche, UCB, Abbvie; serves as a consultant for Abbvie, Alzprotect, Amylyx, Aprineua, Asceneuron, Bayer, Bial, Biogen, Biohaven, Epidarex, Ferrer, Kyowa Kirin, Lundbeck, Novartis, Retrotope, Roche, Sanofi, Servier, Takeda, Teva, UCB; received honoraria for scientific presentations from Abbvie, Bayer, Bial, Biogen, Bristol Myers Squibb, Kyowa Kirin, Pfizer, Roche, Teva, UCB, Zambon; holds a patent on Treatment of Synucleinopathies (US 10,918,628 B2; EP 17 787 904.6‐1109 / 3 525 788); received publication royalties from Academic Press, Kohlhammer, and Thieme. All other authors have no conflicts to report. Author disclosures are available in the supporting information.

## DIAN consortium member list

**Table:** 

Last name	First name	Institution	Affiliation
Bateman	Randall	Washington University	Washington University School of Medicine in St. Louis
Daniels	Alisha J.	Washington University	Washington University in St. Louis
Courtney	Laura	Washington University	Washington University School of Medicine in St. Louis
McDade	Eric	Washington University	Washington University School of Medicine in St. Louis, Department of Neurology
Llibre‐Guerra	Jorge J.	Washington University	Dominantly Inherited Alzheimer's Network, Department of Neurology, Washington University School of Medicine in St.Louis
Supnet‐Bell	Charlene	Washington University	Washington University in St. Louis, School of Medicine, Department of Neurology
Xiong	Chengie	Washington University	Washington University in St. Louis, School of Medicine
Xu	Xiong	Washington University	Washington University in St. Louis, School of Medicine
Lu	Ruijin	Washington University	Washington University in St. Louis, School of Medicine
Wang	Guoqiao	Washington University	Washington University in St. Louis, School of Medicine
Li	Yan	Washington University	Washington University in St. Louis, School of Medicine
Gremminger	Emily	Washington University	Washington University in St. Louis, School of Medicine
Perrin	Richard J.	Washington University	Department of Pathology and Immunology, Department of Neurology, Knight Alzheimer Disease Research Center, Washington University School of Medicine, St. Louis, MO, USA
Franklin	Erin	Washington University	Department of Pathology and Immunology, Washington University in St. Louis
Ibanez	Laura	Washington University	Department of Psychiatry, Department of Neurology, and NeuroGenomics and Informatics Center
Jerome	Gina	Washington University	Washington University in St. Louis, School of Medicine, Department of Neurology
Herries	Elizabeth	Washington University	Washington University in St. Louis, School of Medicine, Department of Neurology
Stauber	Jennifer	Washington University	Washington University in St. Louis, School of Medicine, Department of Neurology
Baker	Bryce	Washington University	Washington University in St. Louis, School of Medicine, Department of Neurology
Minton	Matthew	Washington University	Washington University in St. Louis, School of Medicine, Department of Neurology
Cruchaga	Carlos	Washington University	1. Department of Psychiatry, Washington University School of Medicine, St. Louis, MO, USA 2. NeuroGenomics and Informatics Center, Washington University School of Medicine, St. Louis, MO, USA
Goate	Alison M.	Mount Sinai	Dept. of Genetics & Genomic Sciences, Dept. of Neuroscience, Ronald M. Loeb Center for Alzheimer's Disease, Icahn School of Medicine at Mount Sinai, New York, NY
Renton	Alan E.	Mount Sinai	Ronald M. Loeb Center for Alzheimer's Disease, Dept of Genetics and Genomic Sciences and Nash Family Dept of Neuroscience, Icahn School of Medicine at Mount Sinai
Picarello	Danielle M.	Mount Sinai	Ronald M. Loeb Center for Alzheimer's Disease, Dept of Genetics and Genomic Sciences and Nash Family Dept of Neuroscience, Icahn School of Medicine at Mount Sinai
Benzinger	Tammie	Washington University	Washington University in St. Louis, Department Radiology
Gordon	Brian A.	Washington University	Washington University in St. Louis, Department Radiology
Hornbeck	Russ	Washington University	Washington University in St. Louis, Department Radiology
Chen	Allison	Washington University	Washington University School of Medicine, St. Louis
Chen	Charles	Washington University	Washington University School of Medicine, St. Louis
Flores	Shaney	Washington University	Washington University School of Medicine, St. Louis
Joseph‐Mathurin	Nelly	Washington University	Washington University School of Medicine, St. Louis
Jarman	Steve	Washington University	Washington University School of Medicine, St. Louis
Jackson	Kelley	Washington University	Washington University School of Medicine, St. Louis
Keefe	Sarah	Washington University	Washington University School of Medicine, St. Louis
Koudelis	Deborah	Washington University	Washington University School of Medicine, St. Louis
Massoumzadeh	Parinaz	Washington University	Washington University School of Medicine, St. Louis
McCullough	Austin	Washington University	Washington University School of Medicine, St. Louis
McKay	Nicole	Washington University	Washington University School of Medicine, St. Louis
Nicklaus	Joyce	Washington University	Washington University School of Medicine, St. Louis
Pulizos	Christine	Washington University	Washington University School of Medicine, St. Louis
Wang	Qing	Washington University	Washington University School of Medicine, St. Louis
Sabaredzovic	Edita	Washington University	Washington University School of Medicine, St. Louis
Smith	Hunter	Washington University	Washington University School of Medicine, St. Louis
Scott	Jalen	Washington University	Washington University School of Medicine, St. Louis
Simmons	Ashlee	Washington University	Washington University School of Medicine, St. Louis
Rizzo	Jacqueline	Washington University	Washington University School of Medicine, St. Louis
Hassenstab	Jason	Washington University	Associate Professor of Neurology and of Psychological & Brain Sciences, Washington University in St. Louis
Smith	Jennifer	Washington University	Department of Neurology, Washington University in St. Louis
Stout	Sarah	Washington University	Department of Neurology, Washington University in St. Louis
Aschenbrenner	Andrew J.	Washington University	Assistant Professor of Neurology Washington University in St. Louis
Karch	Celeste M.	Washington University	Department of Psychiatry, Washington University in St Louis
Marsh	Jacob	Washington University	DIAN Fibroblast and Stem Cell Bank, Washington University in St. Louis
Morris	John C.	Washington University	Washington University in St. Louis, Department of Neurology and the Knight Alzheimer Disease Research Center
Holtzman	David M.	Washington University	Department of Neurology, Hope Center for Neurological Disorders, Knight Alzheimer's Disease Research Center, Washington University in St. Louis
Barthelemy	Nicolas	Washington University	Washington University in St. Louis, School of Medicine, Department of Neurology
Xu	Jinbin	Washington University	Department of Radiology, Washington University in St. Louis
Noble	James M.	Columbia University	Taub Institute for Research on Alzheimer's Disease and the Aging Brain, G.H. Sergievsky Center, Department of Neurology, Columbia University Irving Medical Center
Berman	Sarah B.	University of Pittsburgh	University of Pittsburgh Departments of Neurology and Clinical & Translational Science
Ikonomovic	Snezana	University of Pittsburgh	University of Pittsburgh, Department of Neurology
Nadkarni	Neelesh K.	University of Pittsburgh	University of Pittsburgh, Departments of Medicine (Geriatric Medicine) and Neurology
Day	Gregory	Mayo	Department of Neurology, Mayo Clinic in Florida; Jacksonville, FL
Graff‐Radford	Neill R.	Mayo	Department of Neurology, Mayo Clinic in Florida; Jacksonville, FL
Farlow	Martin	Indiana University	Indiana University School of Medicine
Chhatwal	Jasmeer P.	BWH	Massachusetts General Hospital, Brigham and Women's Hospital, Harvard Medical School
Ikeuchi	Takeshi	Niigata	Brain Research Institute, Niigata University
Kasuga	Kensaku	Niigata	Brain Research Institute, Niigata University
Niimi	Yoshiki	Tokyo	Specially appointed lecturer, Unit for Early and Exploratory Clinical Development, The University of Tokyo
Huey	Edward D.	Butler	Memory and Aging Program, Butler Hospital, Professor, Department of Psychiatry and Human Behavior, Alpert Medical School, Brown University
Salloway	Stephen	Butler	Memory and Aging Program, Butler Hospital, Departments of Psychiatry and Human Behavior and Neurology, Alpert Medical School, Brown University
Schofield	Peter R.	Sydney	1. Neuroscience Research Australia, Sydney, Australia 2. School of Medical Sciences, University of New South Wales, Sydney, Australia
Brooks	William S.	Sydney	1. Neuroscience Research Australia, Sydney, Australia; and 2. School of Medical Sciences, University of New South Wales, Sydney, Australia
Bechara	Jacob A.	Sydney	Neuroscience Research Australia, Sydney, Australia
Martins	Ralph	Perth	Edith Cowan University
Fox	Nick C.	UCL	1. Dementia Research Centre, UCL Queen Square Institute of Neurology, London, United Kingdom 2. UK Dementia Research Institute at UCL, London, United Kingdom
Cash	David M.	UCL	1. Dementia Research Centre, UCL Queen Square Institute of Neurology, London, United Kingdom 2. UK Dementia Research Institute at UCL, London, United Kingdom
Ryan	Natalie S.	UCL	1. Dementia Research Centre, UCL Queen Square Institute of Neurology, London, United Kingdom 2. UK Dementia Research Institute at UCL, London, United Kingdom
Jucker	Mathias	Tubingen	1. German Center for Neurodegenerative Diseases (DZNE) Tübingen, Tübingen, Germany 2. Hertie‐Institute for Clinical Brain Research, University of Tübingen, Tübingen, Germany
Laske	Christoph	Tubingen	1. German Center for Neurodegenerative Diseases (DZNE) Tübingen, Tübingen, Germany 2. Hertie‐Institute for Clinical Brain Research, University of Tübingen, Tübingen, Germany
Hofmann	Anna	Tubingen	1. German Center for Neurodegenerative Diseases (DZNE) Tübingen, Tübingen, Germany 2. Hertie‐Institute for Clinical Brain Research, University of Tübingen, Tübingen, Germany
Kuder‐Buletta	Elke	Tubingen	German Center for Neurodegenerative Diseases (DZNE) Tübingen, Tübingen, Germany
Graber‐Sultan	Susanne	Tubingen	German Center for Neurodegenerative Diseases (DZNE) Tübingen, Tübingen, Germany
Obermueller	Ulrike	Tubingen	1. German Center for Neurodegenerative Diseases (DZNE) Tübingen, Tübingen, Germany 2. Hertie‐Institute for Clinical Brain Research, University of Tübingen, Tübingen, Germany
Levin	Johannes	Munich	1. German Center for Neurodegenerative Diseases, site Munich; 2. Department of Neurology, Ludwig‐Maximilians‐Universität München, Munich, Germany; 3. Munich Cluster for Systems Neurology (SyNergy), Munich, Germany
Roedenbeck	Yvonne	Munich	1. German Center for Neurodegenerative Diseases, site Munich; 2. Department of Neurology, Ludwig‐Maximilians‐Universität München, Munich, Germany; 3. Munich Cluster for Systems Neurology (SyNergy), Munich, Germany
Vöglein	Jonathan	Munich	1. Department of Neurology, LMU University Hospital, LMU Munich, Munich, Germany 2. German Center for Neurodegenerative Diseases (DZNE), Munich, Germany
Lee	Jae‐Hong	Seoul	Asian Medical Center, Seoul, South Korea
Roh	Jee Hoon	Seoul	Korea University College of Medicine, Seoul, South Korea
Sanchez‐Valle	Raquel	Barcelona	Alzheimer's Disease and Other Cognitive Disorders Group, Neurology Service, Hospital Clínic de Barcelona, FRCB‐IDIBAPS, University of Barcelona, Barcelona (Spain)
Rosa‐Neto	Pedro	McGill	Translational Neuroimaging Laboratory, McGill University Research Centre for Studies in Aging, Department of Neurology and Neurosurgery, Psychiatry and Pharmacology and Therapeutics, McGill University, Montreal, Canada
Allegri	Ricardo F.	FLENI/Salta	Department of Cognitive Neurology, Instituto Neurológico Fleni, Buenos Aires, Argentina
Chrem Mendez	Patricio	FLENI	Department of Cognitive Neurology, Institute for Neurological Research Fleni, Buenos Aires, Argentina
Surace	Ezequiel	FLENI	Department of Molecular Biology and Neuropathology, Institute for Neurological Research Fleni, Buenos Aires, Argentina
Vazquez	Silvia	FLENI	Center of Molecular Imaging, Institute for Neurological Research Fleni, Buenos Aires, Argentina
Lopera	Francisco	Medellin	Grupo de Neurociencias de Antioquia (GNA), Facultad de Medicina, Universidad de Antioquia, Medellín, Colombia
Leon	Yudy Milena	Medellin	Grupo de Neurociencias de Antioquia (GNA), Facultad de Medicina, Universidad de Antioquia, Medellín, Colombia
Ramirez	Laura	Medellin	Grupo de Neurociencias de Antioquia (GNA), Facultad de Medicina, Universidad de Antioquia, Medellín, Colombia
Aguillon	David	Medellin	Grupo de Neurociencias de Antioquia (GNA), Facultad de Medicina, Universidad de Antioquia, Medellín, Colombia
Levey	Allan I.	Emory	Goizueta Alzheimer's Disease Research Center, Emory University, Atlanta, GA, USA
Johnson	Erik C.B	Emory	Goizueta Alzheimer's Disease Research Center, Emory University, Atlanta, GA, USA
Seyfried	Nicholas T.	Emory	Goizueta Alzheimer's Disease Research Center, Emory University, Atlanta, GA, USA
Ringman	John	University of Southern California	Department of Neurology, Keck School of Medicine of USC, Universty of Southern California
Fagan	Anne M.	Washington University	Department of Neurology, Washington University in St. Louis
Mori	Hiroshi		Osaka Metropolitan University
Masters	Colin	University of Melbourne	Florey Institute, The University of Melbourne

## Supporting information

Supporting Information

Supporting Information

Supporting Information
