# Supplementary material for: α‐Synuclein seed amplification assay detects Lewy body co‐pathology in autosomal dominant Alzheimer's disease late in the disease course and dependent on Lewy pathology burden
Source: Alzheimers Dement. 2024 Apr 26;20(6):4351–65. doi: 10.1002/alz.13818 (PMC11180868; doi:10.1002/alz.13818)
Supplement: Supplementary file 1 — Supporting Information [file ALZ-20-4351-s003.docx]

**
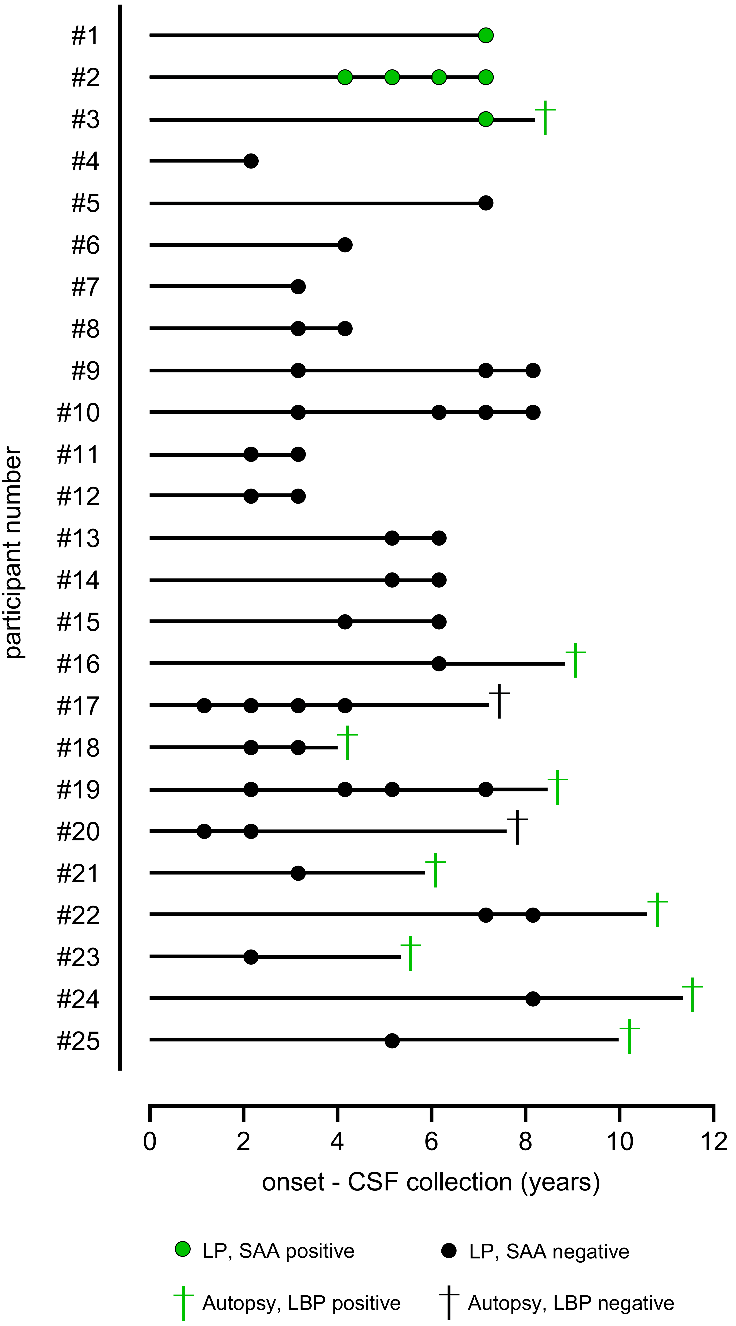
**

**Figure S1**. **Timepoints of CSF collections relative to disease onset in the symptomatic cohort.**

Bullets indicate each CSF sampling (black= SAA negative, green=SAA positive). The solid line represents the symptomatic disease course, while the † the time point of death (black=LBP negative, green= LBP positive). Converters are omitted. Abbreviations: CSF, cerebrospinal fluid; LP, lumbar puncture; SAA, seed amplification assay; LBP, Lewy body pathology.
