# Supplementary material for: α‐Synuclein seed amplification assay detects Lewy body co‐pathology in autosomal dominant Alzheimer's disease late in the disease course and dependent on Lewy pathology burden
Source: Alzheimers Dement. 2024 Apr 26;20(6):4351–65. doi: 10.1002/alz.13818 (PMC11180868; doi:10.1002/alz.13818)
Supplement: Supplementary file 2 — Supporting Information [file ALZ-20-4351-s001.docx]

**
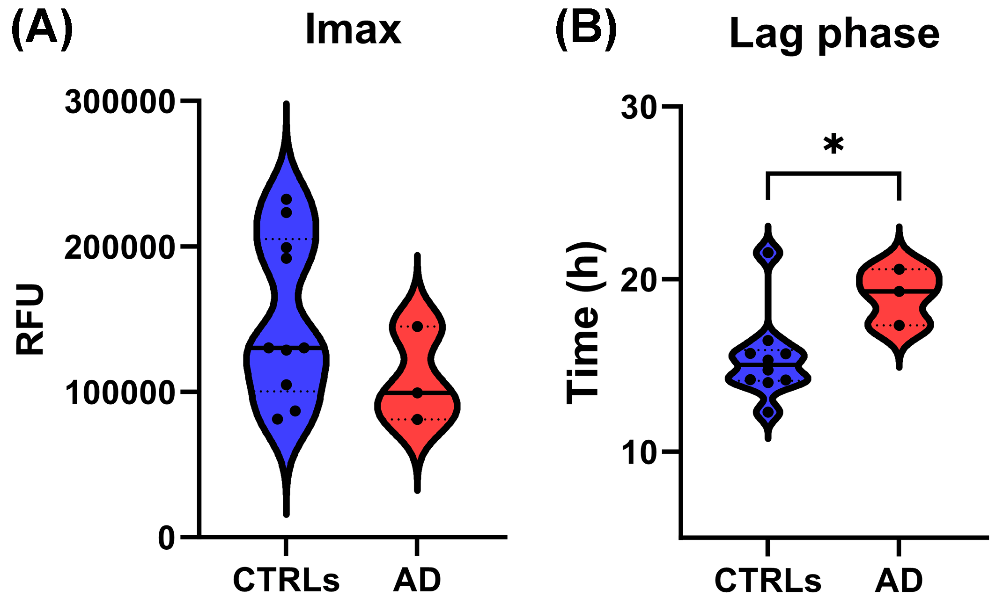
**

**Figure S2**. **Kinetics SAA parameters (Imax and Lag phase) in the two positive controls (see methods) and the three AD patients who showed positive seeding activity in CSF.**

AD cases had a significantly longer Lag phase and a tendency towards lower Imax values than controls. Symbols represent the average values of Imax (A) and Lag phase (B) of positive quadruplicates (positive controls, n=10; AD with positive α-syn SAA, n=3). Abbreviations: Imax, maximum intensity; RFU, relative fluoresce units; CTRLs, controls; α-syn SAA, α-synuclein seed amplification assay. *p ≤ 0.05
